# A Study to Evaluate Accuracy and Validity of the EFAI Computer-Aided Bone Age Diagnosis System Compared With Qualified Physicians

**DOI:** 10.3389/fped.2022.829372

**Published:** 2022-04-08

**Authors:** Chi-Fung Cheng, Ken Ying-Kai Liao, Kuan-Jung Lee, Fuu-Jen Tsai

**Affiliations:** ^1^Big Data Center, China Medical University Hospital, Taichung City, Taiwan; ^2^Ever Fortune.AI Co., Ltd., Taichung City, Taiwan; ^3^Department of Medical Genetics, China Medical University Hospital, Taichung City, Taiwan

**Keywords:** bone age assessment, artificial intelligence, deep learning, concordance correlation coefficient (CCC), clinical practice

## Abstract

**Study Objectives:**

In previous research, we built a deep neural network model based on Inception-Resnet-v2 to predict bone age (EFAI-BAA). The primary objective of the study was to determine if the EFAI-BAA was substantially concordant with the qualified physicians in assessing bone ages. The secondary objective of the study was to determine if the EFAI-BAA was no different in the clinical rating (advanced, normal, or delayed) with the qualified physicians.

**Method:**

This was a retrospective study. The left-hand X-ray images of male subjects aged 3–16 years old and female subjects aged 2–15 years old were collected from China Medical University Hospital (CMUH) and Asia University Hospital (AUH) retrospectively since the trial began until the included image amount reached 368. This was a blinded study. The qualified physicians who ran, read, and interpreted the tests were blinded to the values assessed by the other qualified physicians and the EFAI-BAA.

**Results:**

The concordance correlation coefficient (CCC) between the EFAI-BAA (EFAI-BAA), the evaluation of bone age by physician in Kaohsiung Veterans General Hospital (KVGH), Taichung Veterans General Hospital (TVGH2), and in Taipei Tzu Chi Hospital (TZUCHI-TP) was 0.9828 (95% CI: 0.9790–0.9859, *p*-value = 0.6782), 0.9739 (95% CI: 0.9681–0.9786, *p*-value = 0.0202), and 0.9592 (95% CI: 0.9501–0.9666, *p*-value = 0.4855), respectively.

**Conclusion:**

There was a consistency of bone age assessment between the EFAI-BAA and each one of the three qualified physicians (CCC = 0.9). As the significant difference in the clinical rating was only found between the EFAI-BAA and the qualified physician in TVGH2, the performance of the EFAI-BAA was considered similar to the qualified physicians.

## Background

In pediatrics, the interpretation of bone age can accurately assess the maturity of an individual, and can also be used as a reference for the diagnosis of endocrine disorders in children (1). The well-known manual methods for bone age assessment are Greulich and Pyle (GP method) (2) and Tanner-Whitehouse (TW method) (3). The assessments are based on visual inspection or scoring and are characterized by intra- or extra-observer variability (4, 5). External variability is the difference in judgment standards or differences in the level of interpretation experience among physicians; internal variability is the possible difference in interpretation of the same image by the same physician at different times (6). In addition, the average interpretation time of the GP method in the past study was 1.4 min and TW method was 7.9 min. Both of these methods invisibly increase the time cost of physician visits (7).

In view of the rapid development of artificial intelligence in recent years, image recognition systems developed based on deep learning technology are becoming more and more mature in clinical applications. In the previous research, we introduced the Inception-Resnet-v2 neural network that was pre-trained on ImageNet database, from which to extract features as the basic model (8). At each bone age assessment, the radiologist compares the client’s X-ray image to the GP reference image to assess their bone age and uses this as the ground truth for the model. Using training data from children and adolescents aged 2–18 in Taiwan, the network can predict well when given only the left hand bone X-ray and gender information. The purpose of this AI model is to reduce interpretation errors and actually reduce the complexity, time and cost of the bone age assessment process. The purpose of this research is to use the previously established deep learning model to examine the consistency and effectiveness of this model when it is actually put into clinical application scenarios.

## Materials and Methods

This was a blinded retrospective study. Since all recognizable information had been removed before data collection, no informed consent was required for this study. The qualified physicians who ran, read, and interpreted the tests were blinded to the values assessed by the other qualified physicians and the EFAI-BAA. This study was designed to evaluate the concordance of the EFAI-BAA in assessing bone ages, in comparison to each one of the three qualified physicians.

After the whole included images had been determined, the physicians received the data disk with all included images in and the guidance on how to use the electronic data capture (EDC) system. A physician had to fill in the bone age he/she assessed on the EDC after receiving the data disk. After the bone age corresponding to an image was filled in on the EDC, it might be changed with a rational explanation, and the process was recorded in the EDC. Only after all the physicians finished assessing all the allotted images, can the X-ray images be imported to the EFAI-BAA to get the bone ages inferred by the EFAI-BAA.

### Study Design and Participants

**FIGURE 1:**
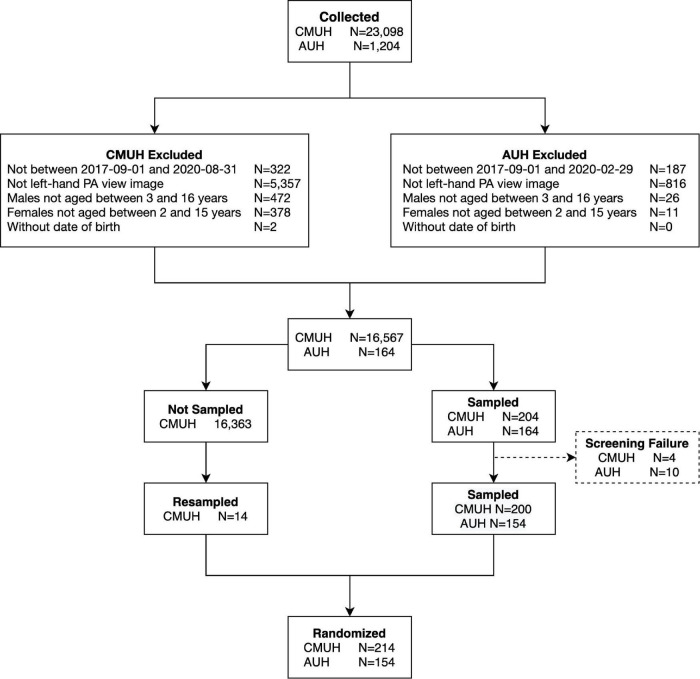
Flowchart of radiographs enrolled in the study.

The study subjects were selected from China Medical University Hospital (CMUH) and Asia University Hospital (AUH). Subjects were enrolled by using the following criteria. Inclusion criteria: (1) Male subjects aged 3 to 16 years old and female subjects aged 2–15 years old at the time of left-hand X-ray PA view image taking. (2) The image quality should be good enough for the physicians to evaluate the bone age. Exclusion criteria: (1) Subjects with skeletal dysplasia. (2) Subjects with congenital anomaly over the hand and wrist. (3) Any severe fracture over the hand and wrist that hindered the determination of the age. (4) Subjects with known malignancy of the left hand. The left-hand X-ray PA view images of male subjects aged 3–16 years old and female subjects aged 2–15 years old at the time when X-ray was taken were retrospectively provided by Medical record department. A total of 368 left-hand X-ray PA view images that met the inclusion/exclusion criteria from these studies were sequentially selected for the proposed study. The flowchart of the subject-selection process is presented in Figure 1.

Three independent certified qualified (with physician license) physicians from three centers in Taiwan, who were not part of the EFAI-BAA development, validation, or clinical study read the left-hand X-ray PA view images. Each of the three qualified physicians was provided with the same set of anonymized left-hand PA X-ray images. They assessed these left-hand X-ray PA view images manually and provide the bone age assessments in the EDC. The same set of left-hand X-ray PA view images were imported to the EFAI-BAA by an independent trained technician for bone age assessment. After the assessments were complete, the results were exported for the statistical analyses.

### Imaging Filtering

In this study, the images were collected retrospectively from CMUH and AUH. A total of 368 DICOM files of left-hand PA view X-ray radiographs were collected (the number of images from either site should not be less than 30%). The information of the subject, which included gender, birth date, and examination date was acquired. At the time when the left-hand X-ray images were taken, the male subjects should be aged 3–16 years old and the female subjects should be aged 2–15 years old.

The X-ray images from Sep 1st, 2017 to Aug 31st, 2020 from CMUH and AUH were queried. The researcher should be used to conduct simple random sampling and provide the order of these random numbers using R (version 3.6.2). The researcher checked the basic information of the subjects including chronological age and gender based on the order and should assign the data to the corresponding age groups.

The expected number in each age group was shown in Table 1.

**TABLE 1:** Summary of baseline characteristics.

Gender	Statistics	Pre-puberty*^a^*	Early and mid-puberty*^b^*	Late puberty*^c^*	Overall
Male	N (%)	94 (25.54%)	66 (17.93%)	30 (8.15%)	190 (51.63%)
	Mean	6.23	11.48	15.12	9.46
	Median	6.34	11.46	15.08	9.27
	SD	1.76	1.19	0.50	3.71
	Min	3.02	9.08	14.12	3.02
	Max	8.93	13.84	15.99	15.99
Female	N (%)	77 (20.92%)	71 (19.29%)	30 (8.15%)	178 (48.37%)
	Mean	4.86	10.05	13.85	8.44
	Median	5.11	10.31	13.73	8.43
	SD	1.56	1.49	0.61	3.68
	Min	2.06	7.04	13.01	2.07
	Max	6.99	12.91	14.92	14.92
Total	N (%)	171 (46.47%)	137 (37.23%)	60 (16.30%)	368 (100.00%)
	Mean	5.61	10.74	14.49	8.97
	Median	5.71	10.84	14.65	8.79
	SD	1.80	1.53	0.85	3.73
	Min	2.06	7.04	13.01	2.06
	Max	8.93	13.84	15.99	15.99

*^a^Pre-puberty (Female: CA 2–7 years old; Male: CA 3–9 years old).*

*^b^Early and Mid-puberty (Female: CA 7–13 years old; Male: CA 9–14 years old).*

*^c^Late Puberty (Female: CA 13–15 years old; Male: CA 14–16 years old). Abbreviation: CA, Chronological Age.*

### Screening

All the included images were burned into a data disk by the research assistant and provided to physicians, to examine the quality of every image. The criteria were (1) Complete left hand and wrist (the distal end of radius and ulna included). (2) The X-ray image of the left-hand PA view. (3) No shadow on the image (such as wearing a ring or a holding fist). (4) The edge of each bone including carpals and metacarpals should be seen and the size of the epiphyseal plate and the degree the epiphyseal plate merged with the bone should be distinguishable.

After the image quality was confirmed, subjects were eligible for enrollment in the study only if they met all the inclusion/exclusion criteria. Subsequently, the research assistant should log in to the EDC system with his/her account and should establish the eCRF for each subject being included after the filtering process. The following information should be entered into the corresponding column: gender, birth date, and the date X-ray taken.

### Re-screening

After the screening process described above, the data amount might be insufficient since the disqualification was sifted. On that occasion, the process was repeated from checking each set of data in the order decided through simple random sampling, assigning the data to the age groups, to the image quality and data qualification screening. The process was repeated until the included amount reached the expected amount.

### Bone Age Assessment

On each included X-ray image, a verification code (ckCode) was marked. Subsequently, the X-ray images along with gender were burned into the data disk, followed by providing two duplicate disks to physicians who participated in this trial. The physicians evaluated the bone age of each image according to the GP method. The physicians logged in to the EDC system with their accounts and passwords. The physicians keyed in the ckCode and corresponding bone age of the image on the eCRF. Only after confirming all the participating physicians had finished evaluating, the included images were imported into the EFAI-BAA by the research assistant to get the bone ages inferred by the medical device for the test.

### Statistical Analysis

The agreement between the EFAI-BAA and each one of the three qualified physicians was assessed using the concordance correlation coefficient (CCC) statistical analysis method (9). The performance of the EFAI-BAA was validated when the concordance criterion between the EFAI-BAA and each one of the three qualified physicians was met. The clinical rating assessed by the EFAI-BAA and the qualified physicians was considered, and the Chi-square test was used to determine the difference in the clinical rating between the EFAI-BAA and each one of the three qualified physicians. The accuracy of the EFAI-BAA compared to each one of the three qualified physicians was calculated as well. The performance of the EFAI-BAA was evaluated by the Root Mean Square (RMS) and Mean Absolute Deviation (MAD) of bone age assessment between the EFAI-BAA and each one of the three qualified physicians. The paired *t*-test was used to compare the mean difference in bone age assessment between the EFAI-BAA and each one of the three qualified physicians. The Bland-Altman plot was created for displaying the difference in bone age assessment between the EFAI-BAA and each one of the three qualified physicians (Supplementary Figures 1–3). For general consideration, descriptive statistics for categorical variables included the number of subjects and percentage; descriptive statistics for continuous variables included the number of observations, mean, SD, median, minimum, and maximum values.

## Results

In this study, the images were collected retrospectively from CMUH and AUH. A total of 368 DICOM files of left-hand PA view X-ray radiographs were collected (the number of images from either site should not be less than 30%). The information of the subject, which included gender, birth date, and date of examination, was acquired. The results of the physicians’ assessments were compared against the bone age assessments by the EFAI-BAA.

The primary endpoint for the study was the bone ages assessed by the EFAI-BAA and the qualified physicians. The analysis result of the primary endpoint was presented in Table 2. The CCC between EFAI-BAA and KVGH (#1) was 0.98 (0.98, 0.99); the CCC between EFAI-BAA and TVGH2 (#2) was 0.97 (0.97, 0.98); the CCC between EFAI-BAA and TZUCHI-TP (#3) was 0.96 (0.95, 0.97).

**TABLE 2:** Differences in the CCC scores (primary endpoint) between three physicians and EFAI-BAA.

Reference	CCC* (95% CI)		
	KVGH (#1)	TVGH2 (#2)	TZUCHI-TP (#3)
EFAI-BAA	0.98 (0.98, 0.99)	0.97 (0.97, 0.98)	0.96 (0.95, 0.97)

*Concordance correlation coefficient (CCC).

The secondary endpoint was the clinical rating assessed by the EFAI-BAA and the qualified physicians. By calculating the 95% interval of the normal bone age distribution by the mean bone age ± 2SD, the bone age assessed would fall within the normal range (normal), out of the upper side of the normal range (advanced), or out of the lower side of the normal range (delayed). The analysis result of the secondary endpoint was presented in Table 3. The number and percentage of “Advanced,” “Normal,” and “Delayed” for EFAI-BAA was 38 (10.33%), 249 (67.66%), and 81 (22.01%), respectively (*p* = 0.6782); for KVGH (#1) was 35 (9.51%), 260 (70.65%), and 73 (19.84%), respectively; for TVGH2 (#2) was 49 (13.32%), 266 (72.28%), and 53 (14.40%), respectively (*p* = 0.0202); and, for TZUCHI-TP (#3) was 41 (11.14%), 259 (70.38%), and 68 (18.48%), respectively (*p* = 0.4855).

**TABLE 3:** Differences in the clinical rating (secondary endpoint) between three physicians and EFAI-BAA.

Site	Clinical rating			Total	*P*-value*^a^*	*P*-value*^b^*
	Advanced	Normal	Delayed			
EFAI-BAA	38 (10.33%)	249 (67.66%)	81 (22.01%)	368 (100.00%)	0.157	ref.
KVGH (#1)	35 (09.51%)	260 (70.65%)	73 (19.84%)	368 (100.00%)		0.6782
TVGH2 (#2)	49 (13.32%)	266 (72.28%)	53 (14.40%)	368 (100.00%)		0.0202
TZUCHI-TP (#3)	41 (11.14%)	259 (70.38%)	68 (18.48%)	368 (100.00%)		0.4855

*^a^Chi-square test of the difference in the clinical rating among the EFAI-BAA and the three qualified physicians.*

*^b^Chi-square test of the difference in the clinical rating between the EFAI-BAA and each of the three qualified physicians.*

The accuracy of the EFAI-BAA was presented in Table 4. The accuracy of EFAI-BAA compared to KVGH (#1) in the pre-puberty, early and mid-puberty, and late puberty group, and the overall age groups was 76.02, 81.02, 93.33, and 80.71%, respectively; the accuracy of EFAI-BAA compared to TVGH2 (#2) in the pre-puberty, early and mid-puberty, and late puberty group, and the overall age groups was 70.76, 86.13, 95.00, and 80.43%, respectively; the accuracy of EFAI-BAA compared to TZUCHI-TP (#3) in the pre-puberty, early and mid-puberty, and late puberty group, and the overall age groups were 66.67, 77.37, 96.67, and 75.54%, respectively.

**TABLE 4:** Accuracy of the EFAI-BAA compared with different sites physicians.

Age group	Accuracy		
	EFAI-BAA vs. #1	EFAI-BAA vs. #2	EFAI-BAA vs. #3
Pre-puberty	76.02%	70.76%	66.67%
Early and mid-puberty	81.02%	86.13%	77.37%
Late puberty	93.33%	95.00%	96.67%
[-1.2pt] Overall	80.71%	80.43%	75.54%

*#1, Kaohsiung Veterans General Hospital (KVGH);*

*#2, Taichung Veterans General Hospital (TVGH2);*

*#3, Taipei Tzu Chi Hospital (TZUCHI-TP).*

The RMS and MAD and paired *t*-test of bone age assessment in each age group were presented in Table 5. The RMS (MAD) between EFAI-BAA and KVGH (#1) in the pre-puberty, early and mid-puberty, and late puberty group, and the overall age groups was 0.81 (0.62), 0.75 (0.60), 1.02 (0.92), and 0.82 (0.66), respectively (*p* = 0.0889); the RMS (MAD) between EFAI-BAA and TVGH2 (#2) in the pre-puberty, early and mid-puberty, and late puberty group, and the overall age groups was 1.22 (0.90), 0.73 (0.56), 0.89 (0.76), and 1.01 (0.75), respectively (*p* < 0.0001); the RMS (MAD) between EFAI-BAA and TZUCHI-TP (#3) in the pre-puberty, early and mid-puberty, and late puberty group, and the overall age groups was 1.19 (0.94), 1.46 (0.88), 0.87 (0.74), and 1.25 (0.89), respectively (*p* = 0.2206).

**TABLE 5:** Root mean square and mean absolute deviation of bone age assessment in each puberty group.

Site	Root mean square (mean absolute deviation)				*P*-value*
	Pre-puberty	Early and mid-puberty	Late puberty	Overall	
EFAI-BAA	ref.	ref.	ref.	ref.	ref.
KVGH (#1)	0.81 (0.62)	0.75 (0.60)	1.02 (0.92)	0.83 (0.66)	0.0889
TVGH2 (#2)	1.22 (0.90)	0.73 (0.56)	0.89 (0.76)	1.01 (0.75)	<0.0001
TZUCHI-TP (#3)	1.19 (0.94)	1.46 (0.88)	0.87 (0.74)	1.25 (0.89)	0.2206

*P-value: paired t-test of bone age assessment for the overall age groups between the EFAI-BAA and each one of the three qualified physicians.

## Discussion

This retrospective study evaluated the accuracy and efficiency of AI system developed for automatic bone age assessment of children in Taiwan. The results show that compared with EFAI-BAA in manually assessed bone age based on the Greulich-Pyle method by three physicians from different hospitals, regardless of gender, this AI model can obtain a highly consistent and accurate bone age assessment by automatically analyzing X-rays of the left wrist.

The bone age assessment of KVGH (#1) was highly consistent with EFAI-BAA in the CCC and the distribution of clinical rating (Tables 2, 3). The bone age assessment of TVGH2 (#2) was averagely higher than that of EFAI-BAA, thus the mean of bone age assessment of TVGH2 (#2) was significantly different from that of EFAI-BAA (Table 5), and the distribution of clinical rating of TVGH2 (#2) was slightly shifted to the grade of “Advanced” (Table 3). Although the divergence of bone age assessment of TZUCHI-TP (#3) was high, TZUCHI-TP (#3) was still similar to EFAI-BAA in the mean of bone age assessment and the distribution of clinical rating (Tables 3, 5), respectively.

Because each lower bound of the two-sided 95% CI of the CCC between the EFAI-BAA and each one of the three qualified physicians was greater than 0.90, the three null hypotheses were all rejected, which meant there was a consistency of bone age assessment between the EFAI-BAA and each one of the three qualified physicians. As the significant difference in the clinical rating was only found between the EFAI-BAA and the qualified physician in TVGH2 (#2), the performance of the EFAI-BAA was considered similar to the qualified physicians.

In recent years, many studies have begun to try to use deep learning methods to assess bone age on left-hand x-ray images (10–16), and a well-trained AI bone age assessment system is as accurate as clinical experts. There was significant intra-individual variability of 0.94 vs. 0.74 years for the GP and TW methods, respectively (7). This variability can be reduced to 0.31 years through EFAI-BAA (8). Clinical diagnostic tools developed by deep learning models are often criticized because they cannot be explained intuitively (black box) (17–19). However, attribute to its excellent interpretation efficiency compared with traditional GP and TW methods, it has been proven to save more interpretation time for physicians (20).

The Greulich-Pyle method is used to assess the maturity of bone age and has been widely used. However, it should be noted that this method is established on Caucasian ethnicity and is highly dependent on the experience of radiologists. It’s prone to cause bias when GP method was applied to different generations, races or specific age groups for bone age assessment (21–26). Similarly, due to this study was a retrospective design, all x-ray images were from the China Medical University Hospital and Asia University Hospital. Therefore, the accuracy of EFAI-BAA has yet to be evaluated in different races or children who were less than 2 years old or over 16 years old. Finally, although there is no statistically significant difference in the assessment between EFAI-BAA and the three clinicians, it does not substitute the doctor’s clinical decision-making, and can only provide the doctor with clinical assistance. EFAI-BAA only predicts the bone age based on the information provided by the images and lacks other clinical information and other physiological factors of the patient.

## Conclusion

In our study, it was shown that there was no statistically significant difference between bone age assessment of EFAI-BAA and three physicians from different sites in Taiwan. In addition, our results show that the AI-based bone age assessment system greatly reduces the time of interpreting bone age by physician compared with the Greulich-Pyle method. It can improve the efficiency of routine clinical examinations without affecting the accuracy of the assessment.

## Data Availability Statement

The original contributions presented in the study are included in the article/Supplementary Material, further inquiries can be directed to the corresponding author.

## Ethics Statement

The studies involving human participants were reviewed and approved by the China Medical University Hospital Institutional Review Board. Written informed consent to participate in this study was provided by the participants’ legal guardian/next of kin.

## Author Contributions

F-JT had the idea and designed the study and responsible for acquisition of data. C-FC, KY-KL, and K-JL analyzed and interpreted the data and provided administrative, technical, logistical or material support. C-FC drafted the article and submitted the manuscript for publication. F-JT and C-FC critically revised the manuscript for important intellectual contents. All authors had the final approval of the manuscript.

## Conflict of Interest

KY-KL and K-JL were employed by Ever Fortune.AI Co., Ltd., Taichung, Taiwan. The remaining authors declare that the research was conducted in the absence of any commercial or financial relationships that could be construed as a potential conflict of interest.

## Publisher’s Note

All claims expressed in this article are solely those of the authors and do not necessarily represent those of their affiliated organizations, or those of the publisher, the editors and the reviewers. Any product that may be evaluated in this article, or claim that may be made by its manufacturer, is not guaranteed or endorsed by the publisher.

## Supplementary Material

Supplementary Figure 1 The Bland-Altman plot for EFAI-BAA vs. KVGH (#1).

Supplementary Figure 2 The Bland-Altman plot for EFAI-BAA vs. TVGH2 (#2).

Supplementary Figure 3 The Bland-Altman plot for EFAI-BAA vs. TZUCHI-TP (#3).
