# Supplementary figures and images for: A Study to Evaluate Accuracy and Validity of the EFAI Computer-Aided Bone Age Diagnosis System Compared With Qualified Physicians
Source: Front Pediatr. 2022 Apr 8;10:829372. doi: 10.3389/fped.2022.829372 (PMC9024098; doi:10.3389/fped.2022.829372)

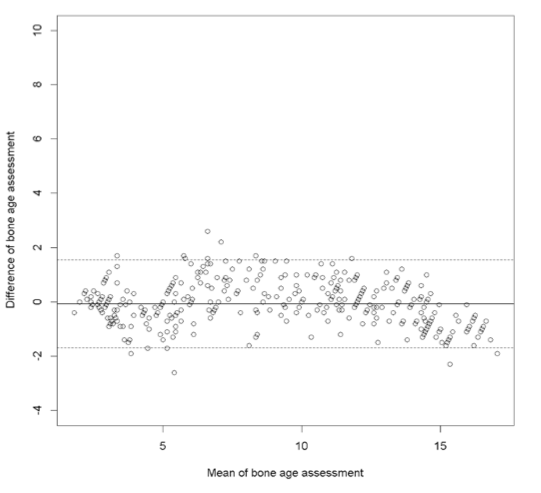

Supplement: Supplementary Figure 1 — The Bland-Altman plot for EFAI-BAA vs. KVGH (#1). [file Image_1.PNG]

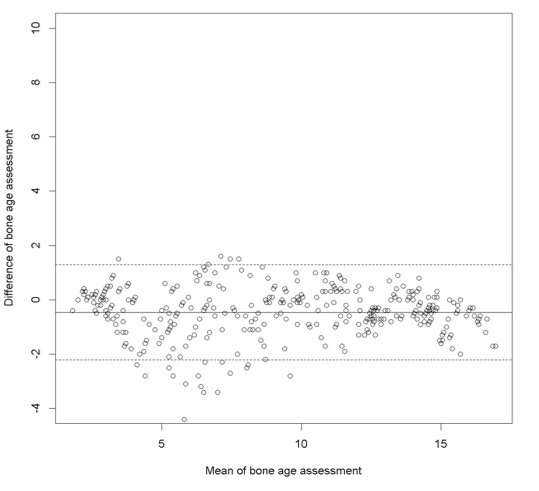

Supplement: Supplementary Figure 2 — The Bland-Altman plot for EFAI-BAA vs. TVGH2 (#2). [file Image_2.PNG]

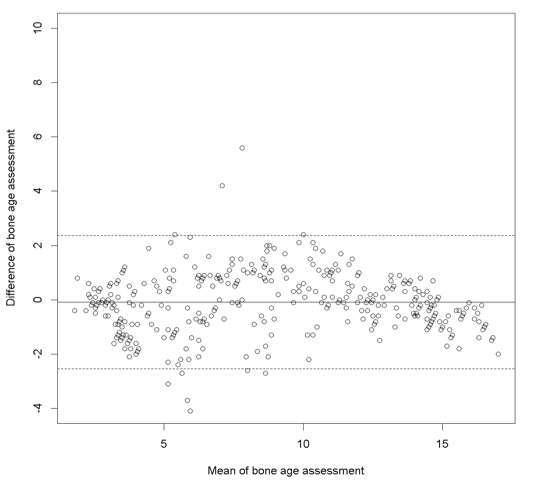

Supplement: Supplementary Figure 3 — The Bland-Altman plot for EFAI-BAA vs. TZUCHI-TP (#3). [file Image_3.PNG]
